# Spatial network characteristics and economic effects of element flow in the Lanxi urban agglomeration

**DOI:** 10.1371/journal.pone.0296496

**Published:** 2024-05-03

**Authors:** Lianchun Zhao, Liu Yang, Xiaoyan Chang

**Affiliations:** 1 College of Economics, Northwest Normal University, Lanzhou, Gansu, China; 2 College of Geography and Environmental Science, Northwest Normal University, Lanzhou, Gansu, China; Huazhong University of Science and Technology, CHINA

## Abstract

The spatial characteristics of element flow and its spillover are important topics in economics, sociology, and geography, and significant to the promotion of the coordinated development of urban agglomerations. To study element flow in the Lanxi urban agglomeration and its effect to economic development, the spatial network characteristics and economic spillover effect were studied using the methods of spatial network analysis, the spatial Durbin model, and spatial effect decomposition. The results showed that (1) the scale of element flow in the Lanxi urban agglomeration is in an unbalanced distribution state, the scale of element flow in Lanzhou and Xining is higher than that in surrounding cities, and the connection between surrounding cities is also higher than that between other cities; (2) the network structure of element flow in the Lanxi urban agglomeration is relatively intensive, with Lanzhou and Xining as the center of element concentration, which indicates an obvious ‘center periphery’ structure, and gradually spreads from the core area to the surrounding areas; and (3) the element concentration level of the Lanxi urban agglomeration has a significant positive spillover effect, which plays a significant role in driving the development of surrounding cities. Other factors, such as the social consumption level, have significant direct effects, whereas the industrial structure and residents’ income have significant direct and spillover effects, and are the main factors that affect the coordinated development of the regional economy.

## 1. Introduction

Urban agglomeration is the aggregation of several cities (towns) that radiate from the central city to the surroundings [1] and a major driver of urbanization. It is an economic growth pole that has great vitality and potential, and has become an important unit in reshaping the spatial pattern of China’s regional economy and promoting coordinated development [2]. As of February 2019, 10 national urban agglomerations were approved in China. They have become important forces in the promotion of the integrated development of major regions and have established a new model of regional development driven by urban agglomeration. China’s urban agglomerations have been greatly developed; however, problems such as simple equilibrium, exclusive dominance, fierce competition, unestablished cooperation, and poor integration remain. Thus, the prospects for regional economic integration and balanced development are not optimistic.

As urban agglomerations have rapidly developed, the ‘temporal and spatial compression effect’ of urban agglomeration has gradually emerged [3], and the research perspective for cities and regions has also begun to shift to ‘flow space’ and its reflected urban networks [4]. The flow and agglomeration of elements are not the only of urban agglomeration processes; there is also the potential interdependence of spatial correlation and economic spillover effects in urban agglomerations between different regions [5]. This reflects the process of competitive cooperation and the uncoordinated development of cities. The interaction and interconnection among cities in an urban agglomeration constantly influence economic growth and spatial evolution, thereby forming a certain spatial network structure and indicating a definite spatial correlation effect [6]. Therefore, research on the spatial network structure and economic spillover effects in urban agglomeration based on element flows is of great importance to the study of the formation of the spatial pattern of urban agglomerations, the process of coordinated development, and the high-quality development path of urban agglomeration. A large amount of recent research in this field has been conducted from the perspective of economic growth [7–9], spillover effects [9, 10], interactions [11–13], spatial correlation structure [14], and spatial evolution [15, 16] using, for example, production functions, gravity models, spatial analysis, and QAP(Quadratic Assignment Procedure) analysis, and so on [17, 18]. However, research on the spatial network structure of element flow and the economic spillover effect of urban agglomerations from the perspective of ‘flow space’ remains inadequate, and research on the mechanism of its coordinated economic development needs to be deepened, particularly in undeveloped urban agglomerations.

The Lanxi urban agglomeration is one of two state-level urban agglomerations in Northwest China. It is not only the ‘key place’ with respect to Tibet and Xinjiang but also a vital link between China and Central Asia, South Asia, and Europe. It plays a critical role in the Yellow River Basin’s high-quality development and has become an important place for promoting the construction of ecological civilization [19]. It is one of the mixed settlements of Han, Tibetan, Mongolian, and Hui ethnic groups. The Lanxi urban agglomeration is situated in the arid and semiarid areas of Northwest China, and has a fragile ecological environment, frequent natural disasters, and a weak economic foundation. The difference in economic development between cities is large because of the effect of these fragile natural conditions and poor economic foundations, and the agglomeration is in a relatively disadvantaged position with respect to the entire nation. In recent years, as the development of the Lanxi urban agglomeration has accelerated, the inadequate and uncoordinated conditions of different cities have become increasingly serious [20]. A scientific analysis of the spatial network structure and economic spillover effect of the Lanxi urban agglomeration can provide theoretical support for the optimization of the network structure and establishment of a long-term mechanism for coordinated regional development. Therefore, using the research method of the spatial Durbin model (SDM) and social network analysis, in this study, the spatial network characteristics of element flow and its economic spillover effect in the Lanxi urban agglomeration are investigated from the perspective of ‘flow space,’ and an attempt is made to clarify the influence mechanism of the spatial structure formation and spatial heterogeneity of the economic development of urban agglomeration. The findings of this study will provide an explanation of the mechanism that drives the economy in the Lanxi urban agglomeration, a prediction of the coordinated economic development and an coordinated economic development in disadvantaged areas of Northwest China, which is of great importance to the implementation of the national strategy of ‘the Belt and Road’ and ‘high-quality development of the Yellow River basin.’

## 2. Study area and data sources

### 2.1 Study area

The Lanxi urban agglomeration is situated at the intersection of Loess Plateau, Inner Mongolia Plateau, and Qinghai-Tibet Plateau in China, and belongs to a multi-ethnic community. The administrative region includes Lanzhou, Xining, Baiyin, Dingxi, Linxia, Hainan, Haibei, Haidong, and Huangnan (location: 34°07′–39°05′ N, 98°05′–105°38′ E, land area of 9.75 × 104 km^2^) [21]. The terrain of the study area is generally high in the west and low in the east, and the region is characterized by a dry climate, low precipitation, semi-arid, and a fragile ecology. It plays an important role in maintaining the prosperity and stability of Northwest China and supporting China’s land ecological security. In 2020, the GDP of the Lanxi urban agglomeration was 641.097 billion yuan and the total regional population was 1.54 × 10^7^. Many ethnic groups, such as Han, Tibetan, Mongolian, Hui, Tu, Sala, Baoan, and Dongxiang, live there.

### 2.2 Data source

Based on the nine cities (states) in the Lanxi urban agglomeration, the main dataset used in this study was collected from the *Gansu Development Yearbook and Qinghai Statistical Yearbook* in 2021. Some data were obtained from the *Statistical Bulletin of National Economic and Social Development* of cities (prefectures) and counties. The map data were obtained from the National Geographic Center, and some missing data were derived by interpolation. The geographical distance between cities (prefectures) was sourced from the GPS positioning distance in the topographic database of the National Basic Geographic Information System published by the State Administration of Surveying and Mapping. Data for the population flow between cities were collected using the Baidu Migration Big Data Platform (https://qianxi.baidu.com/).

## 3. Methodology

### 3.1 Index measurement

In reference to previous research results [22], the scale of capital flow between cities is measured based on the rate of change of the ratio of the capital stock level and the national capital stock level in each city. The calculation formula is as follows:

Capi,t=Ki,tKtKi,t−1Kt−1−1 (1)

where Cap_*i*,*t*_ is the scale of capital flow in city *i* in year *t*, *K*_*i*,*t*_ and *K*_*i*,*t*-1_ are the capital stock levels of city *i* in year *t* and year *t-*1, and *K*_*t*_ and *K*_*t*-1_ are the national capital stock levels in year *t* and year *t-*1, respectively.

Technology flow is characterized by the growth rate of patent applications in each city [23]. The calculation formula is as follows:

$${\mathrm{Tec}}_{i,t}=\frac{T_{i,t}-T_{i,t-1}}{T_{i,t-1}}$$ (2)

where Tec_*i*,*t*_ is the scale of technology flow in city *i* in year *t*, and *T*_*i*,*t*_ and *T*_*i*,*t*-1_ are the number of patent applications in city *i* in year *t* and year *t-*1, respectively.

The population flow is characterized by the migration scale of the population. First, the dataset of daily inflow, outflow, and flow scale between cities in the Lanxi urban agglomeration was obtained from the Baidu Migration Big Data Platform. Then a dataset for one week was randomly selected to assess the tightness of the connection between cities (prefectures). The inter-city flow index is calculated as follows:

Net population flow:

$$M_{net}=M_{in}-M_{out}$$ (3)

where *M*_*net*_ is the net population flow of a city, *M*_*in*_ is the population inflow of a city, and *M*_*out*_ is the population outflow of a city.

The weighted comprehensive index method is used to obtain the scale of element flow between cities (see Table 1). The formula is as follows:

$${\mathrm{Flow}}_{ij}=\sum_{k=1}^{n}M_{ik}\times w_{k}\times Q_{i.j.in}$$ (4)

$$W_{i}=\frac{1-E_{i}}{k-\sum E_{i}}$$ (5)

$$E_{j}=-\ln{\left(n\right)}^{-1}\sum_{i=1}^{n}p_{ij}\ln p_{ij}$$ (6)

pij=Yij∑Yij (7)

where Flow_*ij*_ is the scale of element flow from city *j* to city *i*, *M*_*ik*_ is the flow scale of the *k*-class element of city *i*, *W*_*k*_ is the entropy weight of the *k*-class element, *E*_*i*_ is the entropy of the data, *Y*_*ij*_ is the standardized value of the data, and *Q*_*i*.*j*.*in*_ is the proportion of city *j* in the inflow element of city *i*. Because population flow is an important carrier of element flow between cities, it can reflect the different spatial allocation of production factors, such as capital, information, technology, and labor between different cities [24]. In this study, *Q*_*i*.*j*.*in*_ is characterized by the proportion of population mobility.

**Table 1:** Composite indicators of element flows.

Level 1 indicators	Level 2 indicators	Indicator description	Weight
Element flows	Capital element	The rate of change in the ratio of capital stock	0.2980
	Technical element	Growth rate in the number of patent applications	0.3002
	Population element	The scale of population migration	0.4018

### 3.2 Social network analysis

UCINET social network analysis software was used to analyze the spatial network nodes of element flow, evaluate the status of element flow in each city (prefecture), classify the network centrality of each city (prefecture), and define the ‘role’ and ‘position’ of each city (prefecture) in the entire network [25].

The formula for point degree centrality is expressed as follows:

$$C_{D}=\sum_{j=1}^{g}X_{ij}\left(i\neq j\right)$$ (8)

where *g* is the number of nodes in the network, and $\sum_{j=1}^{g}X_{ij}$ is the number of direct links between node *i* and the other *g*-1 *j* nodes (*i* ≠ *j*).

The formula for network density is expressed as follows:

D=2mn(n−1) (9)

where *m* is the number of connections in the network and *n* is the total number of nodes in the network.

The formula for network centrality is expressed as follows:

$$C_{B}=\sum_{s\neq i\neq t}\frac{n_{st}^{i}}{g_{st}}$$ (10)

where $n_{st}^{i}$ is the number of shortest paths passing through node *i*, and *g*_*st*_ is the number of shortest paths connecting ‘*s*’ and ‘*t*.’

### 3.3 Model construction

Following Atikah [26], the SDM is written as follows:

y=λWy+Xβ+WXδ+ε (11)

where *y* is a dependent variable (GDP per capita), *x* is an independent variable, *Wy* is the spatial lag of the dependent variable, *WX* is the spatial lag of the independent variable, *λ* is the coefficient of *Wy*, *β* is the coefficient of variable *x*, *δ* is the coefficient of variable *wx*, *W* is a spatial weight matrix, and *ε* is a random error.

The main explanatory variable is the scale of element flow. The control variables are the industrial structure (proportion of secondary industry in GDP), the level of residents’ income (per capita disposable income), the level of import and export (total import and export trade), the level of science and technology (expenditure on science and technology), the level of social consumption (total retail sales of social consumer goods), and the urban construction level (space under construction for housing).

The spatial weight matrix of adjacency (*W*_1_) is defined by the Queen adjacency principle:

$$W_{ij}=\{\begin{array}{l} 1,\;\mathrm{if}\;i\;\mathrm{is}\;\mathrm{adjacent}\;\mathrm{to}\;j\\ 0,\mathrm{if}\;i\;\mathrm{is}\;\mathrm{not}\;\mathrm{adjacent}\;\mathrm{to}\;j \end{array}$$ (12)

The spatial weight matrix of geographic distance (*W*_2_) is defined by the inverse of the geographic distance between two cities:

$$W_{ij}=\frac{1}{d_{ij}}$$ (13)

The spatial weight matrix of economic distance (*W*_3_) is defined by the inverse of the distance of the GDP between two cities:

$$W_{ij}=\frac{1}{\left|pergdp_{i}-pergdp_{j}\right|}$$ (14)

### 3.4 Decomposition of spatial spillover effects

The partial differential estimation methods are used to decompose the spatial spillover effects into direct and indirect effects. Among them, the decomposition method proposed by LeSage and Pace [27] is classical. The SDM is converted into the following matrix form:

$$Y={\left(1-\rho W\right)}^{-1}\lambda +{\left(1-\rho W\right)}^{-1}\left(X\beta +WX\delta \right)+{\left(1-\rho W\right)}^{-1}\varepsilon$$ (15)

The partial differential matrix of the ith variable in GDP per capita is

$$\left[\frac{\partial Y}{\partial X_{1i}}\cdots \frac{\partial Y}{\partial X_{ni}}\right]={\left(1-\rho W\right)}^{-1}\left(\begin{array}{ccc} \beta_{i} & \cdots & W_{1n}\delta_{i}\\ \vdots & \ddots & \vdots \\ W_{n1}\delta_{i} & \cdots & \beta_{i} \end{array}\right)$$ (16)

The mean value of the diagonal elements in the transformed matrix represents the direct effect, whereas the mean value of all elements in the upper right of the diagonal of the transformed matrix represents the indirect effect. The direct effect, that is, the local effect, represents the influence of local factor inputs on the economic growth of local cities, whereas the indirect effect, that is, the spatial spillover effect, represents the impact of local factor inputs on the economic growth of neighboring cities.

## 4. Results and analysis

### 4.1 Characteristics of element flow

The analysis of the element flow scale within the city indicated that the element flows of different cities were spatially different, and demonstrated a trend of convection and dispersion. The inflow and outflow scales of Lanzhou were higher than those of other cities, with a net inflow scale index of 0.29, followed by Xining, with a net inflow scale of only 0.1, which indicates that they were net inflow cities. The inflow and outflow of Haibei and Huangnan were relatively small, and the net flow remained essentially unchanged. Although the inflow and outflow of Baiyin, Dingxi, Linxia, and Haidong were relatively large, they were net outflow cities. The total element flow demonstrated that Lanzhou had the largest element flow scale, reaching 2.23, followed by Xining with 1.69. The total element flows of Haidong, Dingxi, Linxia, and Baiyin were relatively large: 1.07, 0.83, 0.67, and 0.60, respectively. Haibei, Huangnan, and Hainan had the lowest total element flows: 0.20, 0.18, and 0.31, respectively. This indicates that Lanzhou and Xining, as the provincial capitals in the region, had scale advantages and their attraction was higher than that of other cities, which also verified that the element flows were closely linked with urban administrative status.

### 4.2 Characteristics of the city connection hierarchy

**Fig 1:**
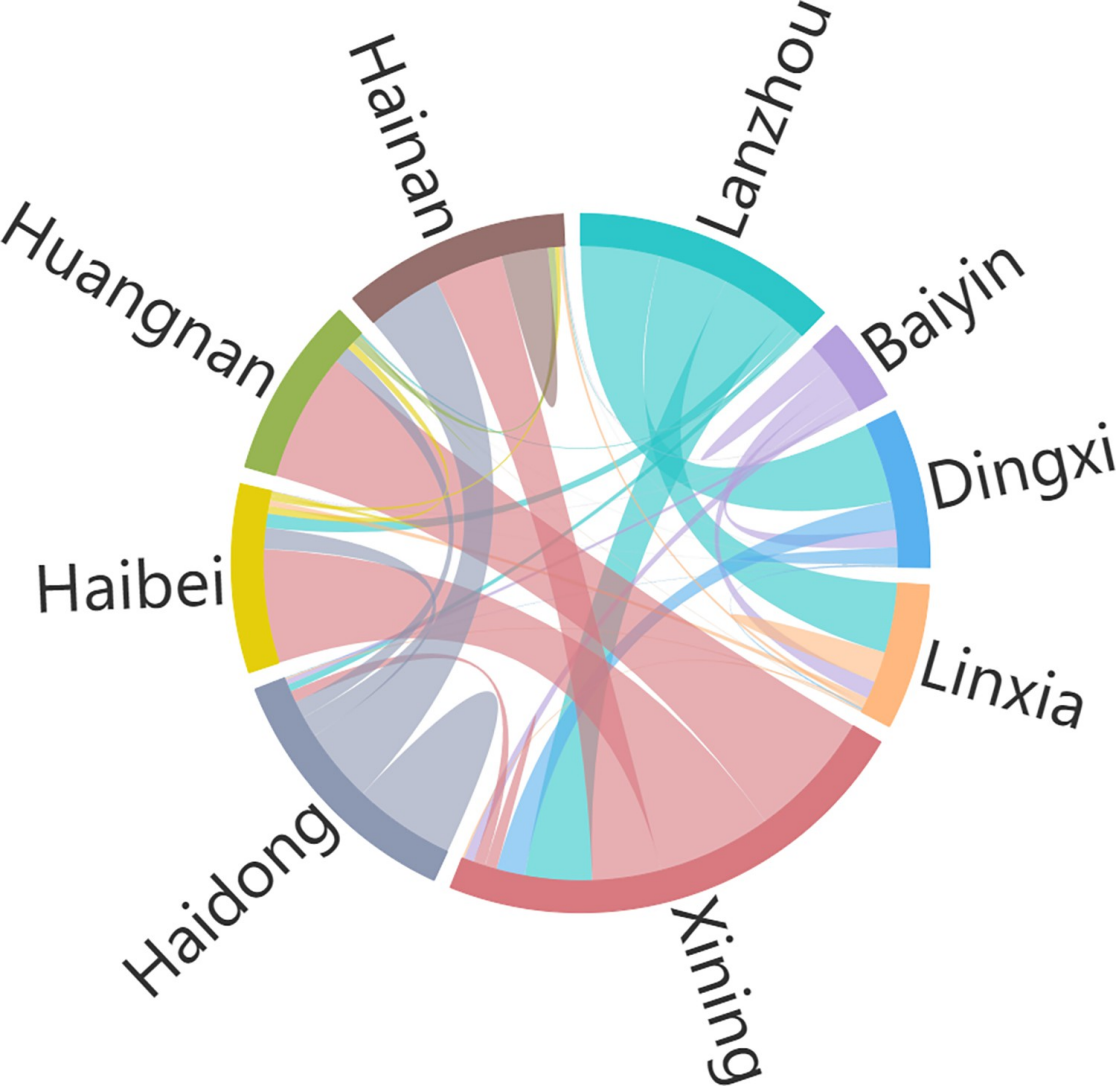
Scale of element flows between cities.

The analysis of element flow intensity between cities indicated (see Fig 1) that the connection between cities varied greatly, and the connection intensity between Lanzhou, Xining, and surrounding cities was higher than that between other cities. The average connection intensity between Lanzhou and the neighboring cities of Haidong, Baiyin, Linxia, and Dingxi was 69.3, and the average connection intensity between Xining and the neighboring cities of Haibei, Hainan and Huangnan was 64.12. Except for Lanzhou, Haidong, and Xining, which were closely connected, with an average connection intensity of 77.5, the connection intensity between cities in the region was low, with an average value of only 1.34. This indicates that spatial correlation exists between the cities in the Lanxi urban agglomeration, and a two-center structure dominated by Lanzhou and Xining has been formed, with an obvious central agglomeration effect.

### 4.3 Network structure of element flow

Based on the social network analysis of UCINET, the network analysis of element flow in the Lanxi urban agglomeration indicated that the network connection within the urban agglomeration was relatively close, with a network density of 0.46. The analysis of network centrality indicated (see Table 2) that the network spatial structure of element flow in the urban agglomeration was characterized by an unbalanced distribution. The degree centralities of Lanzhou, Xining, and Linxia were 5, 4.5, and 2.5, respectively, which were much higher than those of other surrounding cities. The betweenness centralities of Lanzhou and Xining were 14.5 and 11.5, respectively, whereas that of the other cities was only 0. Additionally, the reachability incloseness, and outcloseness of Lanzhou and Xining were also higher than those of other cities. This indicates that Lanzhou and Xining belong to the center of regional element flow, occupy the key position of controlling resource circulation, and are both the ‘source’ and ‘sink’ of element flow. Linxia and Haidong belong to the sub-center of population mobility, thereby playing an important intermediary role. Other cities have low centrality because of their geographical location and low level of economic development. Additionally, Baiyin and Dingxi received more attention than Haibei, Huangnan, and Hainan.

**Table 2:** Network centrality of the Lanxi urban agglomeration.

Region	Degree centrality	Reachability incloseness	Reachability outcloseness	Betweennes centrality
Lanzhou	5.00	32.00	32.00	14.50
Baiyin	2.00	27.59	28.57	0
Dingxi	2.00	27.59	28.57	0
Linxia	2.50	26.67	30.77	0
Xining	4.50	33.33	29.63	11.50
Haidong	2.00	28.57	28.57	0
Haibei	0	11.11	11.11	0
Huangnan	0	11.11	11.11	0
Hainan	1.00	27.59	25.00	0
Network density	0.46			

**Fig 2:**
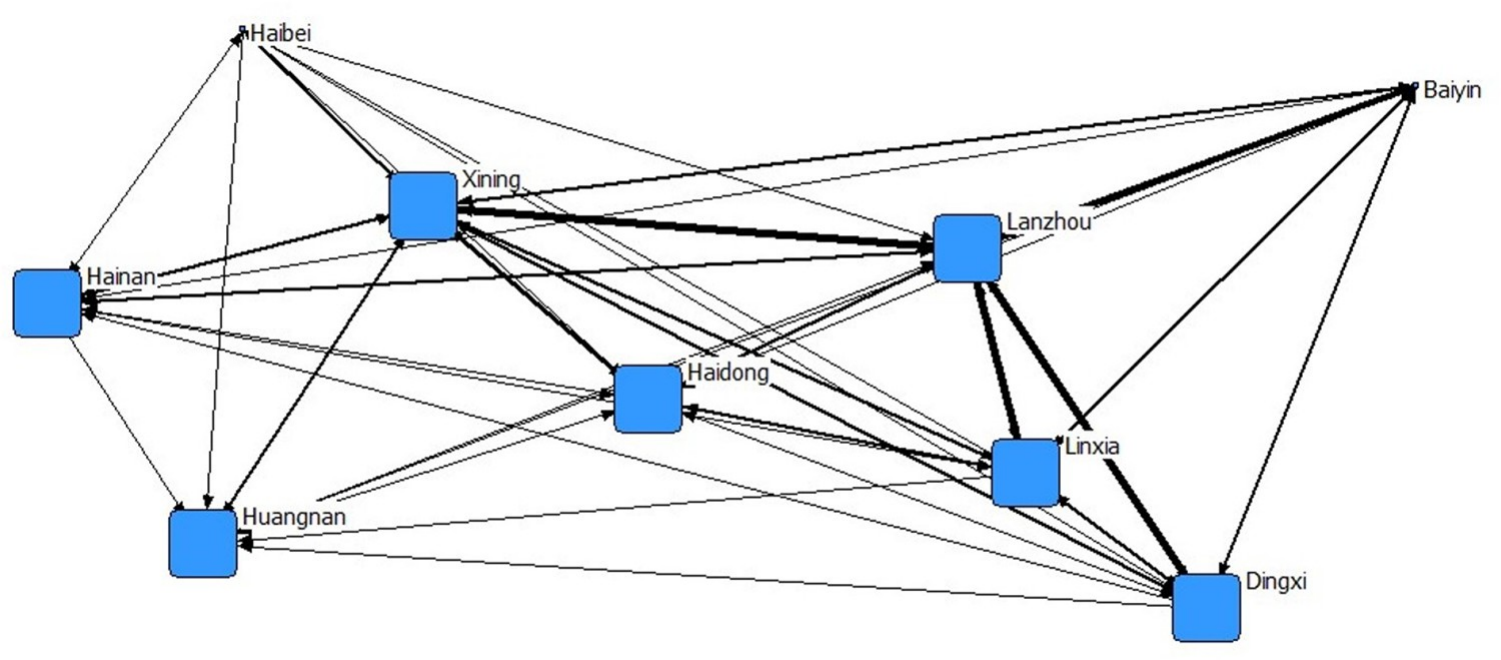
Network structure of the Lanxi urban agglomeration. The thicker the lines, the stronger the connections between cities.

The analysis of the network structure indicated (see Fig 2) that the network structure of the Lanxi urban agglomeration was relatively dense and formed a complex network structure. However, the network structure was mostly around Xining and Lanzhou, and there were relatively few connections between other cities. Again, Lanzhou and Xining were the ‘two cores’ of the Lanxi urban agglomeration, and their ability to control the exchange of elements between other cities was significantly higher than that of other cities(p < 0.05). Other cities were mostly satellite cities of Lanzhou and Xining, and their independence and regional driving ability were relatively weak.

### 4.4 Economic spillover of element flow

#### 4.4.1 Spatial autocorrelation test

The global spatial autocorrelation of the economic development level of each city (prefecture) in the Lanxi urban agglomeration was analyzed. The results demonstrated that the values of the global autocorrelation Moran’s index based on the spatial weight matrix of the economic distance (W3) were positive and significant at the 5% confidence level, which indicates that there is positive spatial autocorrelation in the economic level of each city (prefecture). Therefore, a spatial econometric model can be established for the correlation study.

#### 4.4.2 Spatial econometric model test

To determine whether to use a fixed-effect or random-effect model, Hausman’s test was performed (see Table 3). The result of Hausman’s test indicated that the original hypothesis was rejected (p < 0.01); hence, the fixed-effect model was adopted. The LR test rejected the original hypothesis (p < 0.05); hence, the two-way fixed effect model was adopted. The Wald test rejected the null hypothesis that the double-fixed effect model can degenerate into other effect models s (p < 0.05); hence, finally, the double-fixed effect model was used.

**Table 3:** Hausmann test.

	FE	RE	Difference	S.E.
convergen	0.0199	0.1631	0.1432	0.0705
secondary	0.1831	0.1331	0.0501	0.0907
lnincome	0.4511	0.1362	0.3149	0.1899
lntrade	0.1229	0.0501	0.0728	0.0434
lnscience	0.1052	0.4176	0.3124	0.0776
lnretail	0.4557	0.6745	0.2188	0.1702
lnbuild	0.1662	0.1332	0.0330	0.0320
_cons	0.1344	0.0921	0.0423	0.0906

Note: convergen is the element flow, secondary is the proportion of the total value of the secondary industry, lnincome is per capita disposable income, lntrade is the total import and export trade, lnscience is the expenditure on science and technology, lnretail is the total retail sales of social consumer goods, and lnbuild is the construction area of housing buildings.

#### 4.4.3 Analysis of spatial spillover effects

The decomposition method proposed by LeSage and Pace was used to decompose the spatial spillover effect into a direct effect and indirect effect. The results demonstrated that the scale of element flow, industrial structure level, resident income level, and social consumption level had a significant positive spatial spillover effect (p < 0.1). The indirect effect and the total effect of the convergen variable were significantly positive (p < 0.05), and the direct effect, indirect effect, and total effect of the secondary and lnincome variables were significantly positive (p < 0.1). Additionally, the coefficients of other variables, such as lntrade, lnscience, and lnbuild, were not significant. This indicated that the scale of element flow had a positive impact on the economic development of surrounding cities; the higher the scale, the stronger the driving effect on neighboring cities. The level of industrial structure and residents’ income promoted not only the economic development of the local city but also the economic development of surrounding cities. However, the social consumption level only had a positive impact on the economic development of the region and had no significant impact on neighboring cities. The level of import and export, the level of science and technology and the level of urban construction did not have a significant driving effect on economic development.

#### 4.4.4 Robustness test

The results of models established by different spatial weight matrices may be different. To test the robustness of the result of the selected SDM, the neighboring spatial weight matrix (W1) and the geographic distance spatial matrix (W2) were used for testing (see Table 4). It can be concluded that the estimated coefficients of each variable remained essentially consistent and the coefficient sizes changed slightly. Therefore, this proves that the above analysis results are robust.

**Table 4:** Comparison of the spatial Durbin models of three weight matrices.

Variables	Coefficient (W1)	P-value (W1)	Coefficient (W2)	P-value (W2)	Coefficient (W3)	P-value (W3)
convergen	0.0851	0.228	0.0846	0.183	0.0933	0.077(*)
secondary	0.1322	0.344	0.2285	0.267	0.0971	0.440
lnincome	0.1615	0.365	0.1007	0.493	0.1149	0.438
lntrade	0.0109	0.080(*)	0.0075	0.112	0.0109	0.103
lnscience	0.0004	0.880	0.0009	0.735	0.0064	0.159
lnretail	0.0890	0.354	0.6025	0.009(***)	0.0240	0.830
lnbuild	0.0929	0.439	0.0200	0.801	0.1161	0.039(**)
R-sq	0.9972		0.9968		0.9967	

Note

***, **, * indicate passing the test at 1%, 5%, and 10% significance levels, respectively

## 5. Discussion

### 5.1 Spatial difference of element flow

The scale of element flow in the Lanxi urban agglomeration decreases from east to west in space, and an imbalance exists, which forms a ‘core-edge’ spatial pattern in which the scale of element flow in Lanzhou and Xining is higher than that in surrounding cities (Table 5, Fig 1). The results of this study are consistent with those of previous, related studies [28]. This could be attributable to the uneven distribution of natural, geographical, economic, and other resource endowments in the region. First, the region, which is located in the transition of the first and second steps in China, has a complex topography and diverse geomorphological types. Haibei, Hainan, Huangnan, and other places in the western part of the urban agglomeration belong to the Qinghai-Tibet Plateau, which has a high altitude, large undulating terrain, severe climate, and other natural conditions, which are poor basic conditions for social and economic development, sparse regional population, and relatively slow economic development. Xining, Lanzhou, and other places in the urban agglomeration are located in the river valley basin, which has flat terrain, good basic development conditions, and a relatively concentrated population that forms regional agglomeration centers. It also expands to sub-centers that are geographically close to the center, such as Baiyin, Linxia, Haidong, and other places, which form certain spatial distribution characteristics of a circle. Under the influence of ‘siphonage,’ the resource of the surrounding cities of Lanzhou and Xining are easy to be attracted, thereby forming a ‘dual core’ growth pole of regional development, which leads to the unbalanced spatial distribution of the regional flow scale.

**Table 5:** Scale of element flow.

City	Scale of element inflow	Scale of element outflow	Net element flow	Total element flow
Lanzhou	1.26	0.97	0.29	2.23
Baiyin	0.27	0.33	-0.06	0.60
Dingxi	0.39	0.44	-0.06	0.83
Linxia	0.31	0.36	-0.05	0.67
Xining	0.85	0.84	0.10	1.69
Haidong	0.51	0.56	-0.05	1.07
Haibei	0.10	0.10	0.00	0.20
Huangnan	0.09	0.09	0.00	0.18
Hainan	0.18	0.13	0.05	0.31

### 5.2 Spatial correlation of element flow

As the main form of urbanization in China, urban agglomeration is an important regional unit of regional development and the result of the interaction of agglomeration effects [29]. Social network analysis indicated that the network structure of the Lanxi urban agglomeration tends to develop intensively and forms a network structure with Lanzhou and Xining as the core, and Haidong and Linxia as the secondary core (Table 2, Fig 2), which has a definite spatial correlation. This is related to the geographical proximity of Xining and Lanzhou, in addition to the comprehensive traffic accessibility of the two places. Previous studies in this field have shown that the distribution of cities has obvious traffic directionality, and the evolution of the traffic network is closely related to the development of the cities. The higher the level of cities, the greater the density of their traffic network [30], and the traffic network causes changes to the scale structure and interactions of cities [31, 32]. Lanzhou and Xining, as the political and economic cores of the region, have high town levels, large regional advantages, and close transportation with surrounding regions. Therefore, the intensity of economic ties and the network connections are higher than those of surrounding cities, which have a certain concentration of elements. Particularly in 2018, after the State Council approved the Lanzhou-Xining Urban Agglomeration Development Plan, the connection between cities in the region was strengthened; the production elements, product markets, and financial markets between cities flowed into each other, and the information flow and material flow were constantly coupled. Driven by resource allocation and market competition in the network, the elements not only affected local economic development, but also affected the economic development of neighboring regions. Therefore, this indicates a definite spatial correlation, which is essentially consistent with the research results of other scholars [33].

### 5.3 Analysis of the determinants of economic development

In this study, it was found that element agglomeration has an obvious positive spatial spillover effect, but the local effect is not obvious. This could be because of the network dividend. According to Shy’s ‘network effect’ theory [34], the network has externalities, and it is easy for cities with better network centrality in the element flow to form regional industrial clusters because of their network advantages and strong ability to gather elements. Enterprises in the clusters can promote the exchange or cooperation of elements through network advantages, thus releasing network dividends. Therefore, the higher the scale of element flow in cities, the more likely the efficiency of resource utilization in the surrounding cities is to significantly improve through the ‘resource effect’ of concentration [34], which in turn indicates positive indirect effects. In this study, it was also found that the direct effect, and the spillover effect of the industrial structure level and resident income level are significant. They not only improve the economic development of the region but also promote the economic development of surrounding areas (see Table 6). First, according to the theory of the ‘scale effect’ of agglomeration, it is easy for large urban areas to form industrial agglomerations, thereby improving local and surrounding production efficiency and promoting economic development. Additionally, industrial agglomeration can form a labor pool, intermediate input sharing, and knowledge technology spillover [35], and drive the upgrade of regional industrial structure through resource allocation and market competition. Therefore, the industrial structure level has a positive direct effect and spillover effect. Second, the improvement of residents’ income level plays an important role in stimulating consumption and improving the economic level of local and surrounding areas through the proximity advantage; hence, it has a positive direct effect and indirect effect.

**Table 6:** Spatial spillover effects.

Variables	Direct effect		Indirect (spillover) effects		Total effect	
	Coefficient	P-value	Coefficient	P-value	Coefficient	P-value
convergen	0.0832	0.2620	0.4300	0.0420**	0.3468	0.0480*
secondary	0.2383	0.0251**	0.2226	0.0100***	0.4610	0.0680*
lnincome	0.0983	0.0488**	0.2213	0.0860*	0.3196	0.0100**
lntrade	0.0051	0.8230	0.0137	0.3880	0.0086	0.6490
lnscience	0.0024	0.8620	0.0380	0.2520	0.0404	0.3190
lnretail	0.6124	0.0100**	0.1440	0.6620	0.4684	0.0030***
lnbuild	0.0200	0.8010	0.1121	0.3490	-0.1321	0.4230

Note: ***, **, * indicate passing the test at 1%, 5%, and 10% significance levels, respectively

## 6. Conclusion

Based on the method of social network analysis, in this study, the scale, connection strength, and network relationship of element flow among cities in the Lanxi urban agglomeration were analyzed from the perspective of ‘flow space,’ and the spillover effect of element flow on regional economic development was analyzed using the SDM and spatial effect decomposition model. The results demonstrated that there is spatial difference in the element flow between cities of the Lanxi urban agglomeration, which is in an unbalanced distribution state, and the concentration level of element flow presents an obvious ‘center periphery’ distribution pattern. The scale of element flow and network centrality in Lanzhou and Xining are significantly higher than those in surrounding cities. The connection between surrounding cities is also higher than that between other cities, and forms a complex network structure with Lanzhou and Xining as the ‘core.’ The network structure tends to be intensive. Additionally, the scale of element flow in the Lanxi urban agglomeration has a positive impact on the economic development of surrounding cities, and the driving effect is strong. Other factors, such as the level of industrial structure and residents’ income, not only play an important role in promoting the economic development of this region but also play a certain role in promoting the economic development of surrounding areas. The level of social consumption has a positive impact on the economic development of the region. The level of import and export, the level of science and technology, and the level of urban construction have no significant driving or inhibiting effects on the economic development of the region and neighboring areas. Therefore, the efficiency of element flow can be promoted by continuing to improve the traffic level around the city, improving the efficiency of element flow, and strengthening the construction of service facilities. Additionally, measures could be taken to further optimize the industrial structure, and improve urban employment and residents’ income to promote the coordinated development of the urban agglomeration.

## References

[1] Geddes P. Cities in Evolution. London: Williams & Norgate; 1975.

[2] Qin XY, Qian YS, Zeng JW, Wei XT. Accessibility and Economic Connections between Cities of the New Western Land–Sea Corridor in China—Enlightenments to the Passageway Strategy of Gansu Province. Sustainability. 2022; 14(8), 4445.

[3] Lu DD, Development of Pearl River Delta Metropolitan Group and Pan-Pearl River Delta Economic Cooperation Zone. Economic Geography. 2017; 37(04):1–4.

[4] Tu JJ, Xu GP, Jiang L, Han MT. Characteristics of Flow Spatial Network in the Chengdu-Chongqing Economic Circle. Journal of Southwest University(Natural Science Edition). 2022; 44(01):12–23.

[5] Goodchild M, Haining R, Wise S. Integrating GIS and spatial data analysis: problems and possibilities. International Journal of Geographical Information Systems. 1992; 6(05):407–423.

[6] Tan X, Huang B, Batty M, Li J. Urban Spatial Organization, Multifractals, and Evolutionary Patterns in Large Cities. Annals of the American Association of Geographers. 2021; 111(5):1539–1558. doi: 10.1080/24694452.2020.1823203

[7] Cuberes D, Desmet K, Rappaport J. Urban growth shadows. Journal of Urban Economics. 2021; 123:103334.

[8] Qiao MH. Environmental regulation, technological innovation and the quality of economic development in the Yangtze River Delta city cluster. Statistics and Decision Making. 2022; 38(12):92–95. doi: 10.33422/ijarme.v6i3.1115

[9] Groenewold N, Guoping L, Anping C. Regional output spillovers in China: Estimates from a VAR model. Papers in Regional Science. 2007; 86(01):101–122.

[10] Huang K, Zeng X, Wang H, Zhang J. Industrial Agglomeration of High Technology in Yangtze River Delta City Cluster Based on Knowledge Spillover Spatial Effect Study. American Journal of Industrial and Business Management. 2022; 12(05):1012–1028.

[11] Dziecielski M, Kourtit K, Nijkamp P, Ratajczak W. Basins of attraction around large cities-A study of urban interaction spaces in Europe. Cities. 2021; 119:103366.

[12] Zong HM, Huang Y. Impact of high-speed railroads on regional accessibility and urban interaction patterns in the Chengdu-Chongqing urban agglomeration. Human Geography. 2019; 34(03):99–107,127.

[13] Zhang RJ, Dong HZ. Green development level measurement and spatial correlation structure analysis of cities in Yangtze River Economic Zone. Statistics and Decision Making. 2022; 38(08):118–123.

[14] Gan C, Voda M, Wang K, Chen L, Ye J. Spatial network structure of the tourism economy in urban agglomeration: A social network analysis. Journal of Hospitality and Tourism Management. 2021; 47(06): 124–133.

[15] Sahana M, Hong H, Sajjad H. Analyzing urban spatial patterns and trend of urban growth using urban sprawl matrix: A study on Kolkata urban agglomeration, India. Science of the Total Environment. 2018; 628: 1557–1566. doi: 10.1016/j.scitotenv.2018.02.170 30045573

[16] Bartosiewicz B, Marcinczak S. Urban structure in transition: evidence from Poland, 1983–2011. Regional Studies. 2022; 56(1):36–47.

[17] Zeng JW, Qian YS, Yin F, Zhu LP, Xu DJ. A multivalue cellular automata model for multilane traffic flow under lagrange coordinate. Computational and Mathematical Organization Theory. 2022; 28(2):178–192.

[18] Qian YS, Zeng JW, Wang N, Zhang JL, Wang BB. A traffic flow model considering influence of car-following and its echo characteristics. Nonlinear Dynamics. 2017; 89:1099–1109.

[19] Zeng G, Hu SL. Study on the impact of technological innovation on the green development of cities in the Yellow River Basin. Geoscience. 2021; 41(08):1314–1323.

[20] Gao XC, Yin SK. Measuring the economic efficiency of Lanzhou-Xining urban agglomeration. Urban Issues. 2018; (05):46–52.

[21] Luo ZF, Li XH, Lei DH, Yang ZL. Study on the spatial and temporal variation of quality city construction level and its influencing factors in Lanxi urban agglomeration. Geography of Arid Regions. 2022; 45(01):237–250.

[22] Anas A, Arnott R, Small KA. Urban spatial structure. Journal of Economic Literature. 1998; 36(3):1426–1464.

[23] Yuan QM, Yang QQ, Li J. Evaluation of the Factor Flow Efficiency and Discrepancies among Three Urban Agglomeration. Journal of Dalian University of Technology(Social Sciences). 2018; 39(05):60–68.

[24] Zhang L. Labor Migration, Industrial Transfer and Regional Industrial Agglomeration-An Empirical Study Based on Provincial Panel Data. Collected Essays on Finance and Economics. 2016; (06):3–10.

[25] Buhaug H, Urdal H. An urbanization bomb? Population growth and social disorder in cities.Global Environmental Change. 2013; 23(1):1–10.

[26] White HC, Boorman SA, Breiger RL. Social Structure from Multiple Networks. I. Blockmodels of Roles and Positions. American Journal of Sociology. 1976; 81(04):730–780.

[27] Lesage JP. Introduction to spatial econometrics. New York: Chapman and Hall/CRC; 2009.

[28] Cheng QL, Zhang YF, Song YL. Study on the spatial structure evolution and optimization of Lanxi urban agglomeration. Geographical Research and Development. 2020; 39(02):52–57.

[29] Fang CL. Important progress and prospects of urbanization and urban agglomerations in China in the past 40 years of reform and opening up. Economic Geography. 2018; 38(09):1–9.

[30] Wu N, Shi PJ, Pan X. Analysis of the spatial pattern of the town system in the Jiujia region of Gansu. Geographical Research and Development. 2016; 35(04):85–91.

[31] Zheng W, Du N, Wang X. Understanding the City-transport System of Urban Agglomeration through Improved Space Syntax Analysis. International Regional Science Review. 2022; 45(02): 161–187.

[32] Okumura M, Kobayashi K. The growth of city systems with high-speed railway systems. The Annals of Regional Science. 1997; 31(01):39–56.

[33] Shy O. The Economics of network industries. Cambridge: Cambridge University Press; 2001.

[34] Hirschman AO. The Strategy of Economic Development. New Haven: Yale University Press; 1958.

[35] Ellison G, Glaeser EL, Kerr WR. What Causes Industry Agglomeration? Evidence from Coagglomeration Patterns. The American Economic Review. 2010; 100(03):1195–1213.

